# Effect of Executive Function on Depressive Symptoms in College Students: The Chain Mediating Role of Procrastination Behaviour and Sleep Quality

**DOI:** 10.62641/aep.v53i5.1990

**Published:** 2025-10-05

**Authors:** You Chong, Yan Wang, Ruixue Men

**Affiliations:** ^1^School of Traditional Chinese Medicine, Changchun University of Chinese Medicine, 130117 Changchun, Jilin, China; ^2^School of Marxism, Changchun University of Chinese Medicine, 130117 Changchun, Jilin, China

**Keywords:** students, executive function, procrastination, sleep quality, depressive

## Abstract

**Objective:**

We aimed to analyse the association between executive function (EF) and depressive symptoms (DS) among college students and explore the roles played by procrastination behaviour (PB) and sleep quality (SQ) in this relationship.

**Methods:**

Convenience sampling was adopted in this study. A total of 1618 college students (658 male, 960 female; mean age = 18.987 ± 1.305 years) completed self-administered questionnaires to assess DS and related factors. The Geurten-Questionnaire of Executive Functioning in Chinese College Students, General Procrastination Scale, Pittsburgh Sleep Quality Index and Self-rating Depression Scale were used for the measurements. Normality test, Mann-Whitney U test, Kruskal-Wallis H test, Spearman correlation analysis, multicollinearity tests and Model 6 of the PROCESS macro program were employed for data analysis.

**Results:**

The median score for DS was 47 (interquartile range: 37–58). Place of residence and being an only child were influencing factors of DS among college students (*p* < 0.05). Significant correlations were observed among executive function deficits (EFD), PB, sleep disturbances (SD) and DS in pairwise comparisons (r = 0.380–0.570, *p* < 0.01). Analyses revealed that the total indirect effect (0.178) accounted for 31.34% of the total effect (0.568). The indirect effect of EFD on DS through PB was 0.071 (95% CI = 0.042–0.101). The indirect effect of EFD on DS through SD was 0.072 (95% CI = 0.054–0.093). Meanwhile, the mediating effect through PB and SD was 0.035 (95% CI = 0.023–0.046).

**Conclusion:**

A relatively high prevalence of DS was observed among college students. EFD influenced the DS of college students through the mediating or chain mediating effects of PB and SD, thus providing a theoretical basis for improving the mental health level of college students.

## Introduction

Mental health has become increasingly important in modern society. It manifests as a good and continuous psychological state in which individuals are energetic and mentally fulfilled and have harmonious interpersonal relationships during the growth process; moreover, it is an indispensable part of health [1]. The college stage is a crucial period for individuals to attempt to manage their lives and studies independently and adapt to society. During this period, once an individual’s cognitive function is impaired, which causes difficulty in achieving self-set goals, meeting the eager expectations of their families and fulfilling the academic requirements of the school, they are highly likely to be troubled by depressive symptoms (DS) [2]. DS and their influencing factors have attracted widespread attention in the academic community. The World Health Organisation and numerous professional mental health institutions have emphasised the significance of in-depth research and intervention on DS. DS profoundly reflects an individual’s psychological state and maladaptation to the living environment and encompasses various manifestations, such as prolonged low mood, loss of interest in things that were once enjoyed and significant decline in cognitive function [3]. DS has already become a key indicator of an individual’s psychological crisis, thus seriously affecting the individual’s overall health [4]. Although extensive research has explored DS, most existing studies on the association between executive function (EF) and DS remain at the correlational analysis level. The causal relationship and underlying mechanisms between the two have yet to be clarified fully. Notably, previous research has largely overlooked a key characteristic of college students: this population is in a critical stage of incomplete EF development while facing high-intensity environmental stressors (such as academic competition and social adaptation). The interaction of these two factors may further elevate the risk of DS. Therefore, a chain mediation model that integrates executive function deficits (EFD), Procrastination behaviour (PB), sleep disturbances (SD) and DS is newly constructed, which systematically reveals the cognitive-behavioural-physiological cascade mechanism behind DS formation in college students. These findings provide novel theoretical frameworks and empirical support for mental health education that targets this demographic.

### Executive Function and Depressive Symptoms

Cognitive-behavioural theory emphasises the shaping role of cognition on emotions and behaviours. EF is a high-level cognitive function that directly influences an individual’s cognitive framework and coping strategies towards events by integrating capabilities, such as information processing, planning and decision-making [5]. EF develops rapidly between the ages of 3 and 6 and continues to develop during childhood and adolescence; another relatively significant growth occurs between the ages of 18 and 23, which precisely corresponds to one’s college years [6]. Good EF helps individuals solve difficult situations in reality. For example, individuals with strong EF tend to adopt positive cognitive restructuring strategies, which help to transform negative events into growth opportunities through cognitive reappraisal instead of falling into a cycle of negative thinking [7]. This cognitive flexibility can effectively reduce the incidence of DS. At the same time, EF can be divided into multiple relatively independent executive processing processes. One of its subcomponents, namely, emotional regulation, can serve as a predictor of negative emotions. EFD are clinically common in some mental diseases, including anxiety disorders, depression and obsessive-compulsive disorder [8, 9, 10]. EFD are considered an inducing factor of DS and have been used as a diagnostic criterion for patients with clinical depression in recent years [11, 12]. Therefore, this study proposes the following hypothesis: H_1_: The positive influence of EFD on the DS of college students is significant. A high level of EF among college students entails a decreased possibility of depressive manifestations.

### Mediating Role of Procrastination Behaviour

PB refers to an irrational behaviour in which an individual actively engages in other activities that are unrelated to a task or goal when faced with it, thus resulting in a delay in starting or completing the task. PB is common among college students. This behaviour not only affects their academic development but also leads to emotional experiences, such as anxiety, depression, self-blame and disappointment, thus having a negative impact on the students’ mental health [13]. Individuals with good EF usually possess effective time management skills and self-efficacy. They can plan their time reasonably and have confidence in their ability to complete tasks, thus having decreased PB [14]. By contrast, individuals with EFD may lack effective time management strategies and confidence in their abilities. Most of these individuals have problems regarding distractibility and low self-monitoring ability. They have difficulty following up on a task continuously and are prone to PB. Long-term PB will cause them to encounter several setbacks in their studies and daily lives, thus further decreasing self-efficacy, which can trigger DS. Therefore, the following hypothesis is proposed: H_2_: PB plays a mediating role between EFD and DS among college students.

### Mediating Role of Sleep Quality

Sleep is a fundamental physiological need for individuals; as such, effective sleep serves as the foundation for maintaining physical and mental health [15]. Sleep quality (SQ) is employed to evaluate the quality of sleep. When a series of problems occur in the normal sleep pattern, such issues (such as PB, difficulty falling asleep and insomnia with numerous dreams) will affect the ability to fall asleep, maintain sleep or achieve peaceful sleep, thus leading to daytime sleepiness and fatigue. A large number of research findings indicate that SQ is an important indicator for observing the mental health of college students; furthermore, a mutual influence is observed between SD and DS [16]. According to the emotional-cognitive model of sleep, EF is a superior cognitive function EFD have a negative impact on an individual’s ability to enter and maintain sleep activities [17, 18], thus making people prone to DS. Therefore, the following hypothesis is proposed: H_3_: SD plays a mediating role between EFD and DS among college students.

### Chain Mediating Role of Procrastination Behaviour and Sleep Quality

According to stress system theory, stressors can directly trigger stress responses or act on individuals through intermediary factors, thus causing uncomfortable physical and mental reactions or behaviours and ultimately leading to corresponding outcomes. In this study, EFD is conceptualised as a stressor, which may render individuals who are unable to manage tasks effectively, thus forming a persistent stress. When faced with stress, individuals may regulate their emotions through PB because PB is essentially a coping strategy that actively avoids stress. PB, such as working late into the night and irregular daily routines, directly disrupt sleep architecture, including delayed sleep onset and sleep fragmentation, and consequently leads to SD [19]. Chronic SD can further exacerbate the risk of DS through pathways, such as neuroendocrine disorders and cognitive impairment. Existing research has confirmed that PB includes sleep PB, which is a significant determinant of SD [20, 21]. Therefore, the following hypothesis can be proposed: H_4_: PB and SD play a chain mediating role between EFD and DS among college students.

**Fig. 1.**
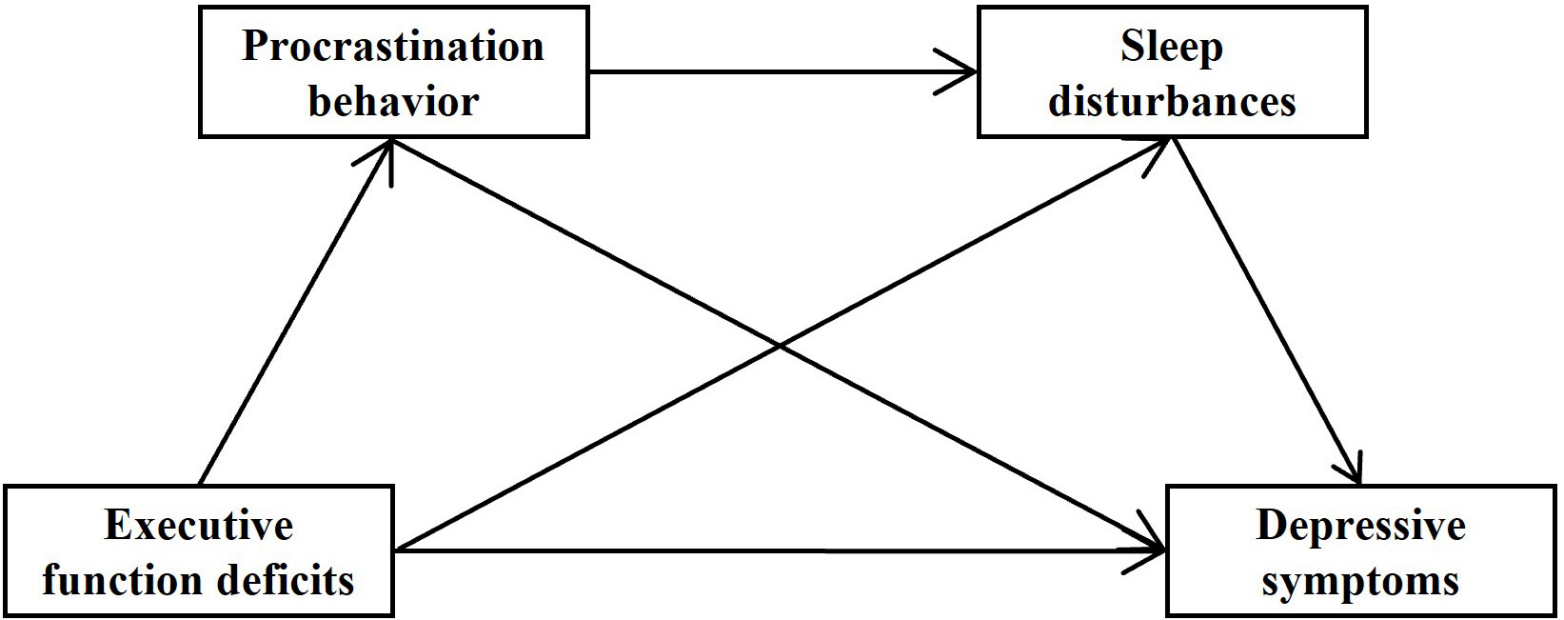
**Hypothetical model of the research**.

Based on previous theories and research, hypotheses are proposed. Additionally, a model is constructed to investigate the relationship between EFD and DS as well as the mediating effects of PB and SD (Fig. 1).

## Methods

### Research Design and Participants

This cross-sectional survey adopted convenience sampling. Monte Carlo power simulations for indirect effects (conducted via MC Power Med) indicated that a minimum sample size of 521 participants was required to achieve 80% statistical power for the primary serial mediation pathway (α = 0.05, two-tailed). In November 2024, general comprehensive undergraduate universities in Jilin Province, Shandong Province, Anhui Province and Gansu Province were selected. College students from different majors and grades in each surveyed university were recruited to participate in the study. The persons in charge of data collection received online training on the administration of the tests. Comprehensive control was exercised over the testing methods, key points and time. Wenjuanxing (an online survey platform) was selected to conduct the survey research with the assistance of the classroom teachers. In addition, classes were taken as units. Before filling out the questionnaires, the persons in charge gave a unified presentation to the respondents, thus enabling the participants to understand the purpose, significance and application of this survey research. In addition, this study adopted an anonymous form. The questionnaires were filled out voluntarily on the premise of confirming the informed consent form. The respondents could abandon the questionnaires at any time during the process. After the questionnaires were collected, the quality of the questionnaires was checked. Those with problems, such as missing answers, very short answering times and identical options, were excluded. The inclusion criteria for the research participants were as follows: (1) full-time undergraduate students, (2) willingness to participate in this study and (3) no previous history of mental illness. The exclusion criteria were as follows: (1) a history of major diseases and (2) a background in psychology education. A total of 1733 questionnaires were distributed in this study. Following the screening principles, such as ‘reverse item inspection’, ‘regular answering inspection’ and ‘exclusion of missing questionnaires’, 1618 valid questionnaires were finally retained with a valid recovery rate of 93.33%. Among them, 658 were male students, while 960 were female students; Moreover, 651, 619, 236, and 112 students were in the first, second, third, and fourth grades, respectively. In addition, 700 students were only children, whereas 918 were not. Meanwhile, 673 students had rural household registration, and 945 had urban household registration. The average age was (18.987 ± 1.305) years old.

### Research Tools

#### Geurten Questionnaire of Executive Functioning in Chinese College Students

This questionnaire was translated and revised by Professor Xue Zhaoxia and colleagues in 2022 based on the Geurten Executive Function Questionnaire [22]. During the revision process, item 22 of the original questionnaire was deleted, thus resulting in a 35-item scale that covered eight dimensions: attention/concentration, working memory, self-monitoring, theory of mind, shifting, impulsivity, planning and emotion regulation. The validity of this questionnaire was verified in the revision study. Exploratory factor analysis showed that eight factors had eigenvalues greater than 1, thus explaining 52.80% of the total variance. Furthermore, the factor loadings of each item ranged from 0.40 to 0.74. Confirmatory factor analysis indicated that the eight-factor model had a good fit (χ^2^/df = 1.93, root mean square error of approximation (RMSEA) = 0.04, comparative fit index (CFI) = 0.92), which was significantly better than the single-factor model (χ^2^/df = 8.62, RMSEA = 0.11, CFI = 0.36). It showed significant positive correlations with the total score of the Behaviour Rating Inventory of Executive Function-Adult Version (r = 0.77) and the total score of the Barratt Impulsiveness Scale (r = 0.71). Moreover, the total score and scores of each factor in the clinical depression group were significantly higher than those in the healthy control group (t = 2.52–13.07, *p* < 0.01, Cohen’s d = 0.43–2.17). The questionnaire demonstrated good reliability and validity in the Chinese cultural context. All items were scored using a four-point Likert scale. The total score of eac dimension was the sum of the scores of the corresponding items. A high total score indicated severe EFD. In this study, the Cronbach’s α coefficient of the scale was 0.87.

#### Short General Procrastination Scale

This scale was derived by Sirois in 2019 through the reduction of the General Procrastination Scale and was revised by Zhang *et al*. [23]. This scale is a single-dimensional test that consists of nine items. All items are scored on a five-point Likert scale. A high total score indicates a strong PB tendency. In this study, the Cronbach’s α coefficient of this scale was 0.78.

#### Pittsburgh Sleep Quality Index

This scale was translated by Professor Liu Xianchen and colleagues from the Pittsburgh Sleep Quality Index developed by Dr. Buysee. The Pittsburgh Sleep Quality Index consists of 18 items and seven component items, which are used to inquire about the SQ of the respondents in the past month. Each item is scored on a scale from 0 to 3, while the total score of the scale ranges from 0 to 21. A high score indicates severe SD. The split-half reliability of this scale is 0.87, while the test-retest reliability is 0.81 [24]. In this study, the Cronbach’s α coefficient of this scale was 0.74.

#### Self-rating Depression Scale

This scale was developed by ZUNG [25] and is one of the most widely applied self-rating depression scales at present. The Self-rating Depression Scale consists of 20 items, which are used to ask the respondents about the frequency of the corresponding situations or feelings that occurred within the past week. A Likert scale ranging from 1 to 4 is adopted for scoring. The raw score is multiplied by 1.25 to calculate the standard score. The total standard score of the SDS ranges from 25 to 100 points. A high total score entails a severe degree of depression. When the total score of the SDS is greater than 53 points, the respondents are considered to have DS. In this study, the Cronbach’s α coefficient of this scale was 0.89.

### Statistical Analysis

Normality of data was evaluated using the Shapiro-Wilk test and Kolmogorov-Smirnov test. Specifically, parametric tests were employed when data met the normality assumption (*p* > 0.05): the independent samples *t*-test for two-group comparisons and a one-way analysis of variance for multigroup comparisons. By contrast, when normality assumptions were not met, nonparametric tests were applied: the Mann-Whitney U test for two groups and the Kruskal-Wallis H test for three or more groups. These tests examine the impact of demographic factors on DS. For analysing relationships among EF, PB, SQ and DS, Pearson correlation was applied under bivariate normality and linearity. Conversely, Spearman’s rank correlation was used if these assumptions were violated. Prior to conducting mediation effect analysis, multicollinearity was assessed using the variance inflation factors (VIFs). This assessment was performed under the following conditions. (1) Predictor variables were either continuous or categorical variables that were dummy-coded for analysis. (2) The sample size exceeded 10 times the number of independent variables. (3) Diagnostics were conducted within the ordinary least squares regression framework. Following the criteria proposed by Hair, a VIF value <5 indicates acceptable collinearity, whereas a VIF ≥5 signals significant multicollinearity issues. Mediation effect analysis was performed using the PROCESS macro program (version 4.2; developer: Andrew F. Hayes, Professor, The Ohio State University, Columbus, Ohio, United States; availability: freely available at https://www.processmacro.org/download.html), specifically Model 6. After controlling for gender, age, place of residence and whether the participants were only children, this model hypothesised that EF was the independent variable, PB was the first mediating variable, SQ was the second mediating variable, and DS was the dependent variable. In this study, the Bootstrap method was applied. The confidence interval (CI) was set at 95%, and the sample size was set at 5000. If the 95% CI did not contain 0, the effect was considered significant.

## Results

### Common Method Bias 

This study controlled common method bias by adopting several procedural measures, including anonymous measurement and adjustment of the direction of some items. After data collection, Harman’s single-factor test was employed to examine the common method bias. The results showed 13 factors with eigenvalues greater than 1. The maximum factor explained 18.41% of the variance, which was less than the critical value of 40%. Therefore, the data did not have severe common method bias.

### Normality Test Results

Shapiro-Wilk (W = 0.949–0.988, *p* < 0.001) and Kolmogorov-Smirnov (D = 0.055–0.103, *p* < 0.001) tests indicated significant deviations from normality for all variables (Table 1).

**Table 1.** **Shapiro-Wilk and Kolmogorov-Smirnov normality test results (N = 1618)**.

Variable	Shapiro-Wilk			Kolmogorov-Smirnov		
	W	df	*p*	D	df	*p*
EFD	0.981	1618	<0.001	0.075	1618	<0.001
PB	0.988	1618	<0.001	0.055	1618	<0.001
SD	0.949	1618	<0.001	0.103	1618	<0.001
DS	0.971	1618	<0.001	0.090	1618	<0.001

Note: EFD, executive function deficits; PB, procrastination behaviour; SD, sleep disturbances; DS, depressive symptoms; W, Shapiro-Wilk statistic; D, Kolmogorov-Smirnov statistic.

### Current Situation of Depressive Symptoms Among College Students and Comparison of Depressive Symptom Scores Across Different Demographic Characteristics

The median score for DS was 47 (interquartile range: 37–58). Among them, the proportions of those without depression, with mild depression, with moderate depression and with severe depression were 61.80%, 31.83%, 5.38% and 0.99%, respectively (Table 2). The results of the Mann-Whitney U test showed that no statistically significant difference existed in depression scores across genders (Z = –0.360, *p* = 0.719). However, place of residence (Z = –2.530, *p* = 0.011 < 0.05) and being an only child (Z = –2.613, *p* = 0.009 < 0.05) were significantly associated with depression scores. The Kruskal-Wallis test indicated that neither age (H = 3.480, *p* = 0.481) nor grade (H = 6.665, *p* = 0.083) had statistically significant effects on depression scores (Table 3). Although gender and age did not show significant effects on depression scores, many previous studies have indicated that gender and age are important factors that influence DS. Such discrepancies may stem from differences in sample characteristics, measurement tools or research environments. Given the potential impact of these variables that were demonstrated in other studies and their prevalence in the research on DS, subsequent studies will control for factors such as gender, age, place of residence and being an only child to explore accurately the effects of other variables on DS among college students.

**Table 2.** **Current status of depression symptoms among college students (N = 1618)**.

Item	Score (Mdn [IQR])	Degree [N (%)]			
		None	Mild	Moderate	Severe
Depression symptoms	47 [37–58]	1000 (61.80)	515 (31.83)	87 (5.38)	16 (0.99)

Note: IQR, Interquartile range.

**Table 3.** **Comparison of DS among college students with different demographic characteristics (N = 1618)**.

Variable		N	Depression symptoms		
			Score (Mdn [IQR])	Statistic	*p*
Gender (MWU)				Z = −0.360	0.719
	Male	658	48 [36–60]		
	Female	960	47 [38–57]		
Age (KWH)				H = 3.480	0.481
	<18	42	51 [37–58.50]		
	18	611	47 [38–58]		
	19	610	48 [38–58]		
	20	230	49 [38–58]		
	>20	125	43 [33–58]		
Grade (KWH)				H = 6.665	0.083
	Freshman	651	47 [38–58]		
	Sophomore	619	48 [38–58]		
	Junior	236	50 [37–58]		
	Senior	112	52 [33–57]		
Place of residence (MWU)				Z = −2.530	0.011
	Rural	673	48 [38–58]		
	Urban	945	47 [37–57]		
Only child status (MWU)				Z = −2.613	0.009
	Only child	700	46 [36–58]		
	Not an only child	918	48 [38–58]		

Note: MWU, Mann-Whitney U test; KWH, Kruskal-Wallis H test; Mdn, Median; IQR, Interquartile range.

### Spearman Rank Correlation Analysis Among Variables and Multicollinearity Diagnosis

The scores of EFD, PB, SD and DS were significantly positively correlated (r = 0.380–0.570, *p* < 0.01). DS increased with the aggravation of EFD, PB and SD (Table 4). Although the dependent variable data did not conform to a normal distribution, the VIF was exclusively employed to detect linear collinearity among predictor variables, which remained unrelated to the distribution pattern of the dependent variable. Consequently, the interpretation of VIF diagnostic results was independent and valid. In all three linear regression models, the VIF values of every predictor variable were substantially below the conventional threshold of 5 (the maximum VIF = 1.596 < 5), thus demonstrating that no severe multicollinearity issues existed among the independent variables in the models (Table 5).

**Table 4.** **Spearman rank correlation analysis among variables (N = 1618)**.

Variable	EFD	PB	SD	DS
EFD	1			
PB	0.570***	1		
SD	0.380***	0.392***	1	
DS	0.543***	0.437***	0.428***	1

Note: ***p < 0.001; EFD, executive function deficits; PB, procrastination behaviour; SD, sleep disturbances; DS, depressive symptoms.

**Table 5.** **Multicollinearity diagnosis results (N = 1618)**.

Outcome	Predictors	Unstandardised Coefficients		Standardised Coefficient	t	*p*	Multicollinearity Diagnosis	
		β	SE	β			Tolerance	VIF
PB	EFD	0.582	0.020	0.582	28.762	<0.001	1.000	1.000
R = 0.582		R^2^ = 0.339		Adjusted R^2^ = 0.338			F = 827.238	
SD	EFD	0.261	0.028	0.261	9.491	<0.001	0.661	1.512
	PB	0.230	0.028	0.230	8.340	<0.001	0.661	1.512
R = 0.437		R^2^ = 0.191		Adjusted R^2^ = 0.190			F = 190.317	
DS	EFD	0.396	0.024	0.396	16.268	<0.001	0.626	1.596
	PB	0.116	0.024	0.116	4.817	<0.001	0.634	1.577
	SD	0.267	0.021	0.267	12.493	<0.001	0.809	1.236
R = 0.634		R^2^ = 0.402		Adjusted R^2^ = 0.401			F = 362.019	

Note: EFD, executive function deficits; PB, procrastination behaviour; SD, sleep disturbances; DS, depressive symptoms.

### Test of the Chain Mediating Effect

**Fig. 2.**
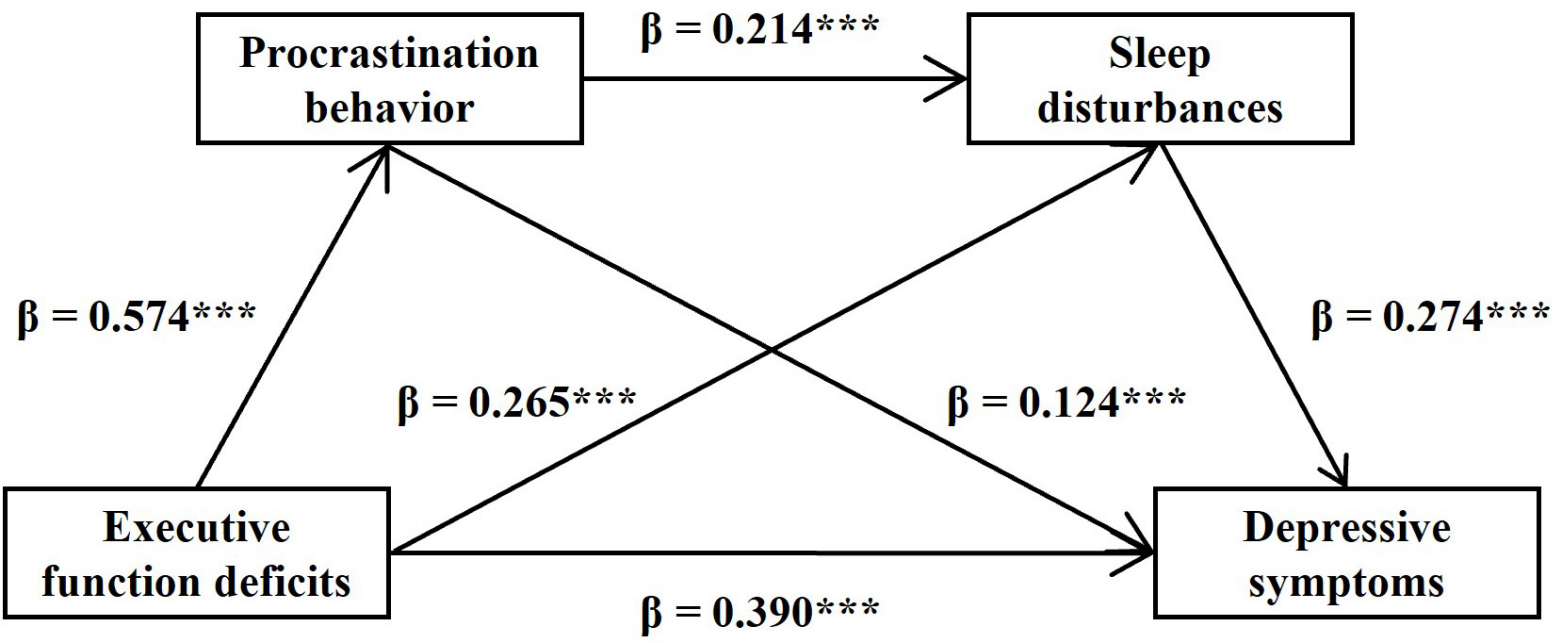
**Model of chain mediating effect**. Note: ***p < 0.001.

The results of the mediating effect analysis showed that the direct effect of EFD on DS was significant (β = 0.390, *p* < 0.001). EFD significantly and positively predicted PB (β = 0.574, *p* < 0.001) and SD (β = 0.265, *p* < 0.001). In addition, PB significantly and positively predicted SD (β = 0.214, *p* < 0.001) and DS (β = 0.124, *p* < 0.001). Meanwhile, SD significantly and positively predicted DS (β = 0.274, *p* <0.001). These results are presented in Fig. 2 and Table 6.

**Table 6.** **Analysis of chain mediation model**.

Outcome	Predictors	β	SE	t	R	R^2^	F
PB	Gender	0.183	0.043	4.270***	0.594	0.352	175.298***
	Age	0.001	0.016	0.030			
	Only child status	0.134	0.044	3.051**			
	Place of residence	0.072	0.044	1.649			
	EFD	0.574	0.020	28.531***			
SD	Gender	0.167	0.048	3.496***	0.447	0.199	66.885***
	Age	−0.008	0.018	−0.462			
	Only child status	0.058	0.049	1.181			
	Place of residence	0.053	0.049	1.080			
	EFD	0.265	0.027	9.657***			
	PB	0.214	0.028	7.713***			
DS	Gender	−0.151	0.041	−3.664***	0.64	0.41	159.557***
	Age	−0.032	0.015	−2.087*			
	Only child status	0.013	0.042	0.302			
	Place of residence	−0.100	0.042	−2.398*			
	EFD	0.390	0.024	16.100***			
	PB	0.124	0.024	5.122***			
	SD	0.274	0.021	12.798***			

Note: *p < 0.05, **p < 0.01, ***p < 0.001; EFD, executive function deficits; PB, procrastination behaviour; SD, sleep disturbances; DS, depressive symptoms.

The direct predictive effect of EFD on DS among college students was significant. PB and SD played a partial mediating role between EFD and DS among college students (Table 7). The research results showed that the total indirect effect (0.178) accounted for 31.34% of the total effect (0.568). Among them, the indirect effect of EFD on DS through PB was 0.071 (95% CI = 0.042–0.101), thus accounting for 12.50% of the total effect (0.568). The indirect effect of EFD on DS through SD was 0.072 (95% CI = 0.054–0.093), thus accounting for 12.68% of the total effect (0.568). Meanwhile, the mediating effect through PB and SD was 0.035 (95% CI = 0.023–0.046), thus accounting for 6.16% of the total effect (0.568).

**Table 7.** **Estimates of total effect, direct effect and indirect effect**.

Structural paths	Standard coefficients (effect value/β)	Effect size	95% CI	
			Lower	Upper
Direct effect	0.390	68.66%	0.343	0.438
Indirect effect 1	0.071	12.50%	0.042	0.101
Indirect effect 2	0.072	12.68%	0.054	0.093
Indirect effect 3	0.035	6.16%	0.023	0.046
Total indirect effect	0.178	31.34%	0.145	0.211
Total effect	0.568		0.528	0.608

Note: EFD, executive function deficits; PB, procrastination behaviour; SD, sleep disturbances; DS, depressive symptoms; CI, confidence interval; direct effect, EFD → DS; indirect effect 1, EFD → PB → DS; indirect effect 2, EFD → SD → DS; indirect effect 3, EFD → PB → SD → DS.

## Discussion

We examined the current situation of DS among college students, the association between EF and DS as well as the mediating effects therein. The results indicated that the college student group had relatively high levels of depression (the proportion of mild depression was 31.83%, that of moderate depression was 5.38%, and that of severe depression was 0.99%). College students living in urban areas had lower depression levels than those in rural areas, which might be attributed to urban areas’ better economic conditions (reducing students’ economic stress) and family education. Only children had lower depression levels than children who had siblings possibly because only children receive more family support and have stronger stress-coping abilities. The high level of DS in college students directly affected their academic performance and mental health. Therefore, educators should attach importance to regulating college students’ negative emotions and improving their mental health. Mediation analysis revealed that EFD influenced DS through the mediating or chain mediating effects of PB and SD.

### Relationship Between Executive Function and Depressive Symptoms

This study focuses on the specific group of college students who are faced with multiple challenges, such as academic pressure and identity transformation. College students need to adapt to the autonomous learning mode of universities within a short period. The difficulty and quantity of courses have increased significantly, while the expansion of the social circle has also brought new pressures in dealing with interpersonal relationships [26]. Negative emotions triggered by situations such as academic setbacks and social isolation that may occur in college life will further interfere with EF, weaken its ability to regulate emotions and exacerbate DS [27]. Our findings confirm a close relationship between the EF and DS of college students. EFD significantly and positively predicted the DS of college students (β = 0.390), thus verifying Hypothesis H_1_. This outcome is consistent with the existing research findings [28]. EF encompasses multiple key components, such as working memory, cognitive flexibility and inhibitory control, which play a core role in an individual’s cognition, emotion regulation and behavioural decision-making [29]. Individuals with good EF can efficiently process various types of information in their studies and life, flexibly respond to various changes, inhibit impulsive behaviours and effectively adapt to the environment and maintain a positive emotional state. When facing difficulties and setbacks, they can use their strong working memory to address problems, find solutions through cognitive flexibility, avoid being trapped in negative emotions and exhibit a low level of DS. From the perspective of dual-process theory, EF belongs to System 2 (the slow-thinking system), which is responsible for rational decision-making and self-control. Depressive tendencies may originate from the excessive activation of automatic negative thinking in System 1 (the fast-thinking system). When EFD, the monitoring and inhibitory ability of System 2 over System 1 declines, thus making individuals likely to be dominated by negative emotions, such as anxiety and self-blame. Therefore, a vicious cognition-emotion cycle is formed.

### Mediating Role of Procrastination Behaviour

Results demonstrated that PB played a mediating role between EFD and DS (β = 0.071), thus verifying Hypothesis H_2_. General procrastinators may exhibit PB because of EFD [30]. They may have working memory deficits, which cause difficulty in concentration. Thus, starting and completing tasks become challenging. They may also experience a decline in emotional regulation ability, which exacerbates the impact of negative emotions, such as anxiety, on their attention (e.g. repeatedly worrying about the consequences of failure). As a result, cognitive resources will shift from task completion to emotional internal consumption. They will eventually fall into symptoms, such as helplessness and depression. Good EF may enable individuals to have a positive attitude when facing difficult situations. With the support of the cognitive processing system, their abilities in reflection and reasoning are strengthened [31]. Moreover, their problem-solving abilities are exercised. They are also less likely to exhibit PB and are likely to gain a sense of achievement and fulfilment. Combined with the stress cognition model, EFD reduces individuals’ perceived controllability of task stress, thus prompting them to avoid stressors through PB. However, task accumulation caused by PB further activates stress hormones (such as cortisol). Long-term accumulation leads to emotional exhaustion, thus increasing the risk of depression.

### Mediating Role of Sleep Quality

Analyses indicated that SD also played a mediating role between EFD and DS (β = 0.072), thus verifying Hypothesis H_3_. Previous studies have shown that EFD is positive correlated with SD [32]. When EFD, individuals show weakened attentional control, thus causing difficulty in suppressing the disordered operation of thoughts at night. Meanwhile, the decline in working memory and inhibitory control abilities creates challenges for them to calm emotions through various strategies, such as cognitive reappraisal [33]. These cognitive dysregulations significantly increase the individual’s cognitive arousal level, thus leading to excessive nighttime brain activity, which interferes with sleep initiation and maintenance and causes a decline in SQ. Long-term SD trigger abnormal cortisol secretion rhythms. This dysregulation not only directly impairs the prefrontal cortex’s regulation of the limbic system, which weakens the brain’s emotional regulation ability, but also prevents the brain from completing neurometabolic repair processes because of insufficient high-quality sleep. This scenario exacerbates the decline in emotional regulation function. Consequently, individuals are prone to negative emotions, such as restlessness and anxiety. Over time, persistent negative emotional experiences and neurophysiological changes significantly increase the risk of developing DS.

### Chain Mediating Role of Procrastination Behaviour and Sleep Quality

We observed that PB and SD played a chain mediating role between EF and DS (β = 0.035), thus verifying Hypothesis H_4_. EFD, including working memory deficits, ineffective self-regulation and insufficient impulse inhibition, makes individuals vulnerable to interference from short-term temptations, such as using mobile phones, or negative emotions, such as anxiety, thus leading to difficulties in task initiation or increased PB [34]. EFD can also exacerbate the tendency to procrastinate by reducing self-efficacy (e.g., I can’t complete anything on time). College students with PB often stay up late because of unfinished tasks (sleep PB) or have difficulty falling asleep because of the anxiety caused by PB (e.g., fear of being criticised by teachers for unfinished tasks), which significantly reduces SQ. SD can directly induce symptoms, such as anxiety and depression, through multiple physiological, psychological and behavioural mechanisms.

### Comparative Analysis of Differences in Mediating Effects

In this study, the single mediating effects of PB and SD on the total indirect effect were significantly higher than the chain mediating effect, thus accounting for 12.50% and 12.68% of the total indirect effect, respectively, compared with 6.16% for the chain mediating effect. The EFD → PB → DS pathway exhibits immediacy. For example, college students with EFD may procrastinate in completing homework. The short-term backlog of tasks can generate negative emotions, thus leading to a stable effect size. The EFD → SD → DS pathway relies on physiological cumulative effects. However, the causal relationship is clear. Thus, the effect size is also stable. The EFD → PB → SD → DS pathway requires the sequential satisfaction of three links: EFD leads to PB, PB leads to SD, and SD lead to DS. However, not all PBs trigger SD (e.g., some students may complete tasks efficiently through last-minute efforts without affecting sleep). In addition, the impact of sleep on depression varies individually (e.g., some individuals have high tolerance to short-term sleep deprivation). The conditional dependence of multiple links dilutes the chain effect. This finding is consistent with the results of parallel studies on smartphone distraction and depression. In the parallel study, rumination and social withdrawal showed a significant chain mediating effect (total indirect effect = 58.64%, chain effect = 7.65%). Both studies showed that the single mediating effects (e.g., PB accounting for 12.50% and SD accounting for 12.68% in this study; rumination accounting for 33.71% and social withdrawal accounting for 17.28% in the parallel study) were consistently higher than the chain mediating effects [35]. This outcome indicated that direct behavioural or physiological pathways might be more stable than sequential cascade pathways.

### Educational Intervention Recommendations

This study reminds educators to attach importance to the impacts of college students’ EF, PB and SQ on DS. Universities can reduce the risk of depression among college students and enhance their mental health level by offering a practical course on time management and emotion regulation, which should include scenario-based simulations to train students in execution skills, such as goal decomposition and priority ranking. Meanwhile, regular mindfulness meditation sessions and group psychological counselling activities should be organised to help students relieve stress. For PB, learning mutual-aid groups supervised by academic tutors can be established. Additionally, themed class meetings on the analysis of PB psychology should be held to guide students in identifying the root causes of their PB. Furthermore, universities can launch a ‘Sleep Health Month’ campaign. This initiative may include meditation and relaxation courses and a distribution of sleep-aid supplies to help students develop regular sleep habits.

### Limitations

This study has certain limitations. Firstly, this work is a cross-sectional study, which restricts the inference of causal relationships. In the future, prospective studies are needed to test the findings of this study. Secondly, all the scales used in this study are self-reported scales, which may lead to social desirability bias. In the future, additional objective methods can be adopted for data collection. Thirdly, potential confounding factors (such as academic stress, socioeconomic status and social support) and comorbid mental disorders that may confuse DS (such as anxiety) were not fully considered in this study. These factors may affect the validity and interpretation of the results. Future studies should exclude such potential confounding factors. Fourthly, Harman’s single-factor test has limitations. Future research should adopt more robust methods. Fifthly, the sample was only sourced from four provinces and used a convenience sampling method, thus posing risks regarding limited geographical coverage and selection bias. Additionally, the sample lacked specific demographic information about students (e.g., major and family background), which restricts the generalisability and representativeness of the research results. In the future, the sampling scope should be expanded to multiple regions nationwide. Rigorous random sampling methods should also be employed. In addition, specific student demographic information (e.g., major, family background and race/ethnicity) should be collected to improve the representativeness and generalisability of the research results.

## Conclusion

DS has a relatively high prevalence among college students. In addition, EFD influences the DS of college students through the mediating or chain mediating effects of PB and SD. The study has uncovered the cognitive-behavioural-physiological cascading mechanisms behind the development of DS in college students. Interventions that target the enhancement of EF, reduction of PB and promotion of healthy sleep habits hold great potential for improving mental health among college students.

## Data Availability

The datasets used and/or analysed during the current study were available from the corresponding author on reasonable request.

## Abbreviations

EF, executive function; EFD, executive function deficits; PB, procrastination behaviour; SQ, sleep quality; SD, sleep disturbances; DS, depressive symptoms; CI, confidence interval; VIF, variance inflation factor; MWU, Mann-Whitney U test; KWH, Kruskal-Wallis H test; Mdn, median; IQR, interquartile range.
